# Lipid-derived radical-trapping antioxidants suppress ferroptosis

**DOI:** 10.1093/lifemeta/loae008

**Published:** 2024-03-06

**Authors:** Ruoxi Zhang, Guido Kroemer, Daolin Tang

**Affiliations:** Department of Surgery, UT Southwestern Medical Center, Dallas, TX 75390, United States; Centre de Recherche des Cordeliers, INSERM U1138, Equipe labellisée–Ligue contre le cancer, Université Paris Cité, Sorbonne Université, Institut Universitaire de France, Paris 75006, France; Metabolomics and Cell Biology Platforms, Gustave Roussy Cancer Campus, Villejuif 94800, France; Institut du Cancer Paris CARPEM, Department of Biology, Hôpital Européen Georges Pompidou, Assistance Publique–Hôpitaux de Paris, Paris 75015, France; Department of Surgery, UT Southwestern Medical Center, Dallas, TX 75390, United States

**Ferroptosis, characterized by lipid peroxidation-mediated cell demise, is governed by a nuanced interplay of lipid species influencing its vulnerability. Two recent publications in *Nature* discovered 7-dehydrocholesterol, a cholesterol precursor, as a radical-trapping antioxidant that can suppress ferroptosis, thereby presenting a novel metabolic target to improve ferroptosis-related cancer therapy.**

Oxidative stress arises from the generation of reactive oxygen species and other free radicals, which cause damage to vital biomolecules, such as lipids, proteins, and DNA. This damage disrupts cellular function and integrity, ultimately precipitating cell death. Among various forms of oxidative cell death, the pharmacological induction of ferroptosis is emerging as an attractive anticancer strategy to suppress tumor growth, particularly in traditionally drug-resistant cancer cells [1].

**Figure 1:**
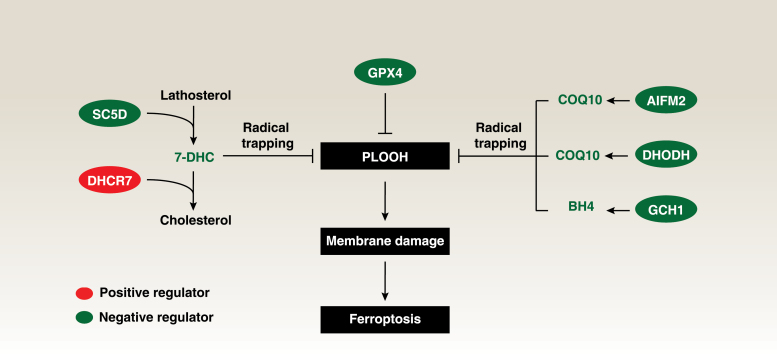
7-DHC acts as a radical trapping antioxidant protecting ferroptosis. 7-DHC serves as an intermediate metabolite in the distal cholesterol biosynthesis pathway, synthesized by SC5D and metabolized by DHCR7 for cholesterol synthesis. In the context of ferroptosis, 7-DHC functions as a radical-trapping antioxidant, inhibiting the initiation of lipid peroxidation and consequent cell death. This antioxidant activity complements other GPX4-dependent and GPX4-independent surveillance systems, such as the AIFM2–COQ10 axis, DHODH–COQ10 axis, and GCH1–BH4 axis. PLOOH, phospholipid hydroperoxide; BH4, tetrahydrobiopterin; COQ10, coenzyme Q10.

The molecular mechanism of ferroptosis is heterogeneous and plastic [2]. Classical ferroptosis is initiated by iron-dependent Fenton reactions and subsequent lipid peroxidation [3]. This process results in oxidative damage to membrane lipids, particularly polyunsaturated fatty acid (PUFA)-containing phospholipids, leading to the accumulation of toxic lipid products that induce membrane dysfunction, rupture, and cell demise. Conversely, cells can deploy multiple levels of defense mechanisms to clear or limit oxidative damage and lipid peroxidation products, especially phospholipid hydroperoxides (PLOOHs) (Fig. 1). One such defense mechanism is mediated by the master antioxidant enzyme, glutathione peroxidase 4 (GPX4), which neutralizes lipid peroxides by reducing PLOOH into phospholipid alcohols. Several GPX4-independent enzymes, including apoptosis-inducing factor mitochondria-associated 2 (AIFM2, also known as ferroptosis suppressor protein 1), dihydroorotate dehydrogenase (DHODH), and GTP cyclohydrolase 1 (GCH1), can produce metabolites with free radical-trapping antioxidant (RTA) activity in a context-dependent manner (Fig. 1). RTAs operate by chemically reacting with free radicals, effectively trapping them, and thus averting further damage. Unlike traditional antioxidants, which primarily function by donating electrons to stabilize free radicals, RTAs physically capture and neutralize radicals by forming stable complexes with them.

While understanding the lipid metabolism of ferroptosis remains a central topic in this field, the existence of endogenous lipids or metabolites with RTA activity remains uncertain. However, two recent complementary studies conducted by Freitas *et al*. and Li *et al*. published in *Nature* have illuminated this area by suggesting an unexpected role of the cholesterol precursor, 7-dehydrocholesterol (7-DHC), as a natural suppressor of ferroptotic cancer cell death through RTA activity [4, 5] (Fig. 1).

By employing a genome-wide CRISPR–Cas9-based screen, both research teams independently pinpointed a crucial enzyme involved in cholesterol synthesis, 7-DHC reductase (*DHCR7*), as a key gene regulating ferroptosis induced by GPX4 inhibitors, such as RAS-selective lethal 3 (RSL3) and ML210, in Pfa1 or HEK293T cells, respectively. *DHCR7* deficiency led to the accumulation of its substrate, 7-DHC, consequently suppressing ferroptosis. Subsequent reconstitution of DHCR7 abolished 7-DHC accumulation and restored cell sensitivity to ferroptosis without affecting the response to other agents inducing cell death. Additionally, Li *et al*. identified several proteins involved in distal cholesterol biosynthesis—methylsterol monooxygenase 1 (MSMO1), cytochrome P450 family 51 subfamily A member 1 (CYP51A1), cholestenol delta-isomerase (also known as emopamil binding protein), and sterol-C5-desaturase (SC5D)—as suppressors of ferroptosis. Deletion of these genes selectively increased cell susceptibility to ferroptosis, a lethal effect that was effectively reversed by inhibitors of ferroptosis, but not inhibitors of apoptosis or necroptosis, underscoring the specific involvement of the cholesterol synthesis pathway in ferroptosis.

Assessing the impact of cholesterol on ferroptosis, both groups demonstrated that deletion of *SC5D* or *DHCR7* had minimal effects on cholesterol levels. Direct addition of cholesterol to sterol-free cell culture medium exerted little influence on ferroptosis, leading to the conclusion that cholesterol does not directly regulate ferroptosis. Moreover, the deletion of *SC5D* or *DHCR7* failed to alter the expression of established ferroptosis regulators or the abundance of metabolites, providing additional support for this conclusion.

Considering that SC5D catalyzes the conversion of lathosterol to 7-DHC, the major intermediate metabolite converted to cholesterol by DHCR7, the authors explored the role of 7-DHC in ferroptosis regulation. Deletion of *SC5D* in *DHCR7*-deficient cells reversed the suppressive effect of *DHCR7* deficiency on ferroptosis, while *DHCR7* deletion failed to suppress ferroptosis in *SC5D*-deficient cells, emphasizing the essential role of 7-DHC production in ferroptosis regulation. Treatment with DHCR7-selective inhibitors or cariprazine increased 7-DHC levels and suppressed ferroptosis in wild-type cells, but not in *SC5D*- or *DHCR7*-deficient cells or cells with defective 7-DHC biosynthesis. Exogenous supplementation of 7-DHC also efficiently protected against ferroptosis. These findings collectively suggest that 7-DHC is a potent ferroptosis suppressor, meaning that its abundance can dictate ferroptosis sensitivity.

Exploring the mechanism underlying 7-DHC’s ferroptosis suppression, the authors focused on its impact on phospholipid peroxidation. Both groups observed that 7-DHC treatment or *DHCR7* deletion blocked RSL3-induced lipid peroxidation, while depletion of 7-DHC-enhancing enzymes promoted lipid peroxidation. Moreover, 7-DHC and *DHCR7* deletion blocked mitochondrial lipid peroxidation induced by GPX4 and DHODH inhibitors, whereas *SC5D* deletion promoted such induction. In addition, 7-DHC became detected on both plasma and mitochondrial membranes, with its deficiency reducing phospholipid peroxidation without affecting intrinsic phospholipid levels, suggesting a broad anti-ferroptotic effect across membranes, particularly during the early stages of ferroptosis.

Given 7-DHC’s unique conjugated 5,7-diene structure, known for its high oxidizability, both teams hypothesized that this structure enabled 7-DHC to absorb radicals, thereby preventing phospholipid autoxidation. Utilizing fluorescence-enabled inhibited autoxidation assay, they found that 7-DHC, but not other sterols, blocked phospholipid autoxidation. Ergosterol, sharing a similar structure with 7-DHC, also exhibited potent activity against phospholipid autoxidation and conferred ferroptosis inhibition. Furthermore, induction of ferroptosis increased the oxidation product of 7-DHC, 3β, 5α-dihydroxycholest-7-en-6-one, with suppression of phospholipid peroxidation corresponding to 7-DHC intervention in the radical chain reaction. These findings suggest that oxidation of unsaturated B-ring sterols, including 7-DHC, protects cells from phospholipid peroxidation induced by free radicals by diverting the peroxidation pathway from phospholipids.

With its protective effect against ferroptosis, targeting 7-DHC holds promise for advancing cancer treatment and managing ferroptosis-related diseases. Investigating its impact on cancer cell vulnerability to ferroptosis, Freitas and colleagues observed rare *DHCR7* mutations in individuals with Burkitt’s lymphoma or neuroblastoma, leading to 7-DHC accumulation and ferroptosis resistance, indicating a more aggressive phenotype. Similarly, Li *et al*. found that blocking 7-DHC synthesis increased cancer cell susceptibility to ferroptosis, with neuroblastoma cells exhibiting high 7-DHC levels. Pharmacological inhibition of 7-DHC biosynthesis induced ferroptosis and inhibited neuroblastoma cell growth *in vitro* and *in vivo*. Moreover, elevated 7-DHC levels promoted melanoma cancer cell metastasis. Li *et al*. also demonstrated the protective effect of elevated 7-DHC levels against ischemia-reperfusion injury and ferroptosis in kidneys, suggesting its potential therapeutic application in ferroptosis-related diseases outside of the realm of oncology.

In conclusion, these two recent *Nature* papers mark a significant advance in our comprehension of the antioxidant role of 7-DHC as an intracellular regulator of ferroptosis. Tumor cells exploit their anti-ferroptotic activity to evade cell death, insinuating that reducing 7-DHC levels through pharmacological intervention could be a promising avenue for cancer therapy. Conversely, increased levels of 7-DHC may offer protection against ischemia-reperfusion injury. Notably, trazodone, an inhibitor of 7-DHC synthesis by DHCR7, is used to treat patients with mental health conditions, including depressive disorders, insomnia, anxiety disorders, and sometimes neuropathic pain [6]. Whether such drugs can be repurposed for the attenuation of excessive ferroptosis remains to be investigated.

While these studies undoubtedly propel ferroptosis research, they also raise important new questions. First, both studies did not explore the potential impact of 7-DHC on ferroptosis-related immune responses within the tumor microenvironment. Crafting therapies that selectively target the 7-DHC pathway in cancer cells without disrupting vital cellular processes or causing adverse effects on normal cells pose a formidable challenge [7]. Rigorous evaluation of deficient 7-DHC production on the immune microenvironment may pave the way for innovative strategies in developing cell-specific ferroptosis inducers. Second, elucidating the circumstances under which cancer cells utilize GPX4-dependent or GPX4-independent antioxidant pathways, including those involving 7-DHC, to evade ferroptosis is crucial. Unraveling the upstream signals and potential feedback mechanisms governing distinct downstream pathways holds the key to monitoring ferroptosis sensitivity effectively. Third, beyond its role as a cholesterol precursor, 7-DHC is an essential precursor of vitamin D synthesis. Prior research in CHO-7 (Chinese hamster ovarian cell line) and sterol-resistant CHO cells indicates that *DHCR7* loss leads to 7-DHC accumulation, subsequently boosting vitamin D production [8]. Recent studies also suggest that vitamin D inhibits ferroptosis in various cell types [9]. Furthermore, PUFA-containing cholesterol esters (PUFA-CEs) are believed to be sensitive to lipid peroxidation in the context of lipid flippase solute carrier family 47 member 1 (*SLC47A1*) depletion [10]. Therefore, it is crucial to determine whether the anti-ferroptotic effect of 7-DHC is vitamin D-dependent or involves inhibiting lipid peroxidation on PUFA-CEs. Addressing these questions could enhance our understanding of the role of 7-DHC in the pathophysiology of ferroptosis.
